# Supplementary material for: Recurrent Neural Network Exploration Strategies During Reinforcement Learning Depend on Network Capacity
Source: Comput Brain Behav. 2025 Oct 28;9(2):184–95. doi: 10.1007/s42113-025-00258-4 (PMC13293025; doi:10.1007/s42113-025-00258-4)
Supplement: Supplementary file 1 — (DOCX 289 KB) [file 42113_2025_258_MOESM1_ESM.docx]

**Supplemental Material**

**Table S1** *Group-Level Performance Measures*

| Agents | Cumulative Regret | | Switch % | |
| --- | --- | --- | --- | --- |
|  | *M* | *SE* | *M* | *SE* |
| 48 | 1393.802 | 46.143 | 15.022 | 0.505 |
| 64 | 1378.122 | 46.651 | 15.346 | 0.357 |
| 80 | 1323.295 | 41.295 | 14.741 | 0.391 |
| 96 | 1382.994 | 48.856 | 13.648 | 0.291 |
| 192 | 1333.505 | 59.913 | 13.046 | 0.47 |
| 576 | 1209.349 | 47.249 | 12.895 | 0.41 |
| Humans | 1389.628 | 102.528 | 28.965 | 2.287 |

*Note*. *RNNs*: *N* = 30 observations per group. *Humans*: *N* = 31 observations.

**Table S2** *Number and Percentage of Runs in Which the SM + EDP Model Accounted Best for the Behavior of the RNNs as a Function of Network Capacity*

| No. of hidden units | $\Delta WAIC>0$ | |
| --- | --- | --- |
|  | *n* | *%* |
| 64 | 16 | 18.2 |
| 80 | 25 | 28.4 |
| 96 | 37 | 42 |
| 192 | 43 | 48.3 |
| 576 | 59 | 67 |

*Note*. $\Delta WAIC$ = Difference in WAIC values between the SM + DP and the SM + EDP model; $\Delta WAIC >0$ indicates a superior fit of the SM + EDP model over the SM + DP model.

**Table S3** *Group-Level Model Parameters*

| Agents | $\beta$ | | $\Phi$ | | $\rho$ | | $\alpha_{h}$ | |
| --- | --- | --- | --- | --- | --- | --- | --- | --- |
|  | *M* | *SE* | *M* | *SE* | *M* | *SE* | *M* | *SE* |
| 48 | 0.118 | 0.003 | -0.25 | 0.112 | 16.627 | 0.554 | .647 | .015 |
| 64 | 0.11 | 0.002 | -0.334 | 0.075 | 16.557 | 0.517 | .678 | .017 |
| 80 | 0.118^^[[1]](#footnote-1)^^ | 0.002 | -0.318 | 0.113 | 15.483 | 0.622 | .67 | .017 |
| 96 | 0.128 | 0.004 | -0.462 | 0.126 | 13.543 | 0.683 | .697 | .017 |
| 192 | 0.135 | 0.004 | 0.035 | 0.132 | 15.398 | 0.772 | .794 | .019 |
| 576 | 0.163 | 0.004 | 0.802 | 0.113 | 15.86 | 0.675 | .789 | .019 |
| Humans | 0.168 | 0.01 | 1.479 | 0.308 | 10.904 | 1.097 | .571 | .036 |

*Note*. *RNNs*: *N* = 30 agents per group. *Humans*: *N* = 31 agents. $\beta$: Inverse temperature parameter; $\Phi$: Exploration bonus; $\rho$: Perseveration bonus; $\alpha_{h}$: Habit step-size parameter.


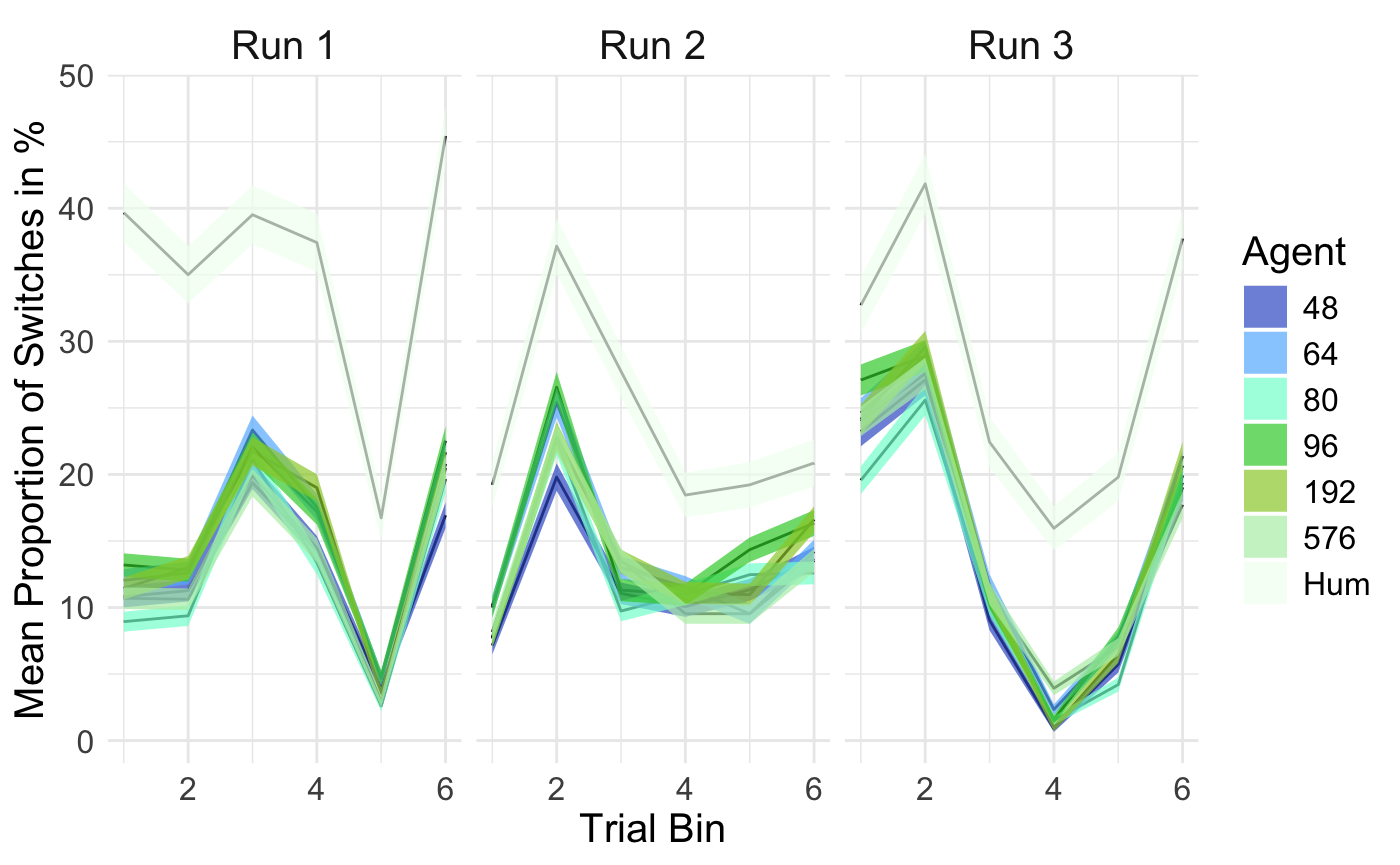


**Figure S1. Similar switch rate dynamics between networks and human learners.** X-axes display trial bins with 50 trials per bin. Lines represent networks grouped by capacity, defined by the no. of hidden units (48-576), along with human learners (Hum). Shaded areas represent SE.

1. *n* = 28 observations [↑](#footnote-ref-1)
